# Grading hypoxic-ischemic encephalopathy in neonatal EEG with convolutional neural networks and quadratic time–frequency distributions

**DOI:** 10.1088/1741-2552/abe8ae

**Published:** 2021-03-19

**Authors:** Sumit A Raurale, Geraldine B Boylan, Sean R Mathieson, William P Marnane, Gordon Lightbody, John M O’Toole

**Affiliations:** 1 Irish Centre for Maternal and Child Health Research (INFANT), University College Cork, Cork, Ireland; 2 Department of Paediatrics and Child Health, University College Cork, Cork, Ireland; 3 Department of Electrical and Electronic Engineering, University College Cork, Cork, Ireland; jotoole@ucc.ie

**Keywords:** electroencephalography, hypoxic-ischemic encephalopathy, time-frequency distribution, convolutional neural network

## Abstract

*Objective.* To develop an automated system to classify the severity of hypoxic-ischaemic encephalopathy injury (HIE) in neonates from the background electroencephalogram (EEG). *Approach*. By combining a quadratic time–frequency distribution (TFD) with a convolutional neural network, we develop a system that classifies 4 EEG grades of HIE. The network learns directly from the two-dimensional TFD through 3 independent layers with convolution in the time, frequency, and time–frequency directions. Computationally efficient algorithms make it feasible to transform each 5 min epoch to the time–frequency domain by controlling for oversampling to reduce both computation and computer memory. The system is developed on EEG recordings from 54 neonates. Then the system is validated on a large unseen dataset of 338 h of EEG recordings from 91 neonates obtained across multiple international centres. *Main results.* The proposed EEG HIE-grading system achieves a leave-one-subject-out testing accuracy of 88.9% and kappa of 0.84 on the development dataset. Accuracy for the large unseen test dataset is 69.5% (95% confidence interval, CI: 65.3%–73.6%) and kappa of 0.54, which is a significant ($P\lt0.001$) improvement over a state-of-the-art feature-based method with an accuracy of 56.8% (95% CI: 51.4%–61.7%) and kappa of 0.39. Performance of the proposed system was unaffected when the number of channels in testing was reduced from 8 to 2—accuracy for the large validation dataset remained at 69.5% (95% CI: 65.5%–74.0%). *Significance.* The proposed system outperforms the state-of-the-art machine learning algorithms for EEG grade classification on a large multi-centre unseen dataset, indicating the potential to assist clinical decision making for neonates with HIE.

## 1. Introduction

Hypoxic-ischaemic encephalopathy (HIE) is a major cause of neonatal mortality and morbidity with an incidence of 3–5 per 1000 births in high income countries [1]. HIE occurs around time of birth due to lack of oxygen (hypoxia) and impaired blood supply (ischemia) in the brain [2]. HIE continues to evolve over time after the primary hypoxic-ischemic (HI) insult [2]. Therapeutic hypothermia has become the standard treatment for neonates with moderate to severe HIE [3, 4]. To be effective however, it should be initiated within 6 h of birth, offering a small time window for treatment.

It is sometimes difficult to recognise which babies would benefit most from therapeutic hypothermia. Current neonatal practice relies on the initial assessment of the infant’s clinical state and other clinical markers to grade the severity of encephalopathy following birth. Murray *et al* [5] have shown that these markers can be unreliable and that early information about brain function would be extremely helpful. Also, sedative drugs can confound clinical assessment [5]. And lastly, the severity of the neonatal EEG grade can provide useful information about long-term outcome which helps the clinical team guide critical management [6].

Electroencephalography (EEG) provides an effective non-invasive tool for monitoring neonatal cortical activity after a HI injury. Neonates with HIE are also at high risk of seizures and the EEG is essential to detect and quantify the seizure burden. Depending on the severity of the insult, the amplitude, frequency, sleep cycling, and continuity can be affected in various ways; in addition, abnormal transient waveforms and seizures may be present [6]. Nonetheless, continuous EEG monitoring and interpretation is difficult, time consuming, and must be assessed by an experienced neurophysiologist. This capability is not always available in most NICUs on a 24/7 basis. Automated grading of the EEG, using computer-based algorithms, has the potential to overcome the practical limitations of reviewing extensive, continuous EEG monitoring in the NICU for all neonates with suspected HIE, giving the attending neonatologist a simple instantaneous evaluation of brain function. This information could help inform not only initial decisions regarding the initiation of hypothermia therapy, but also ongoing care during the period of critical illness and indeed issues concerning outcome.

A number of algorithms have been proposed to grade the EEG for HIE. Stevenson *et al* [7] proposed a method which extracted non-stationary features of the time–frequency distribution (TFD). The TFD was generated on short-duration epochs (64 s with 50% overlap) and features of the instantaneous amplitude and instantaneous frequency were extracted. These features were combined with more standard quantitative EEG features using a multi-class linear discriminant classifier. Matić *et al* [8] proposed a tensor-based approach in which continuous EEG was first adaptively segmented and three features extracted from each segment to build a tensor. This tensor was reduced using multi-dimensional decomposition methods and then combined using a least-squares support vector machine (SVM). Another study by Matić *et al* [9] proposed a dynamic interburst intervals (dIBIs) detection approach combined with multi-class SVM classifier to quantify background EEG dynamics in term neonates with HIE.

Further, Ahmed *et al* [10] used a larger feature set of 55 values from the time, frequency, and information theory domains extracted from an 8 s window (with 50% overlap) of EEG. A Gaussian mixture model (GMM) supervector was constructed from a sequence of these features extracted over 80 s of EEG. An SVM combined these supervectors and the grade was determined across multiple channels and across one hour of EEG recording. Rather than using a large number of generic EEG features, we previously proposed [11] the use of two features of the temporal distribution of inter-burst activity extracted from a burst–interburst detector; these features have clinical relevance as inter-burst interval (IBI) increases with severity of HIE, and is therefore closer to the analysis carried out by the neurophysiologist. A multi-layer perceptron was then used to classify EEG grades. In a recent study, Guo *et al* [12] used multiple time and frequency domain features from long- and short-term segments coupled with an SVM. More recently, we proposed a one-dimensional convolutional neural network (CNN) approach to automate feature extraction from the raw EEG segment to classify EEG grades [13].

These existing automated grading systems employ complex feature sets to extract information from the raw EEG [7, 9, 10, 12], before combining with machine learning methods. These sophisticated and sometimes physiologically meaningful features are needed to capture the wide range of signal characteristics associated with the different grades. Bypassing the feature-based approach and building on our existing work [13], here we apply a deep-learning approach which uses all the information from the EEG signal without the need to design and select features [13–15]. Within the field of deep-learning, CNN have demonstrated state-of-the-art results in many medical image processing tasks [16, 17].

For the one-dimensional EEG data, time–frequency representations have been used in place of two-dimensional images. Spectrograms of the raw EEG have been used as input to CNN models for effective sleep scoring [14, 18–20] and seizure detection [15, 21] tasks, for example. Spectrograms are intuitive time–frequency representations that can be computed efficiently for long-duration signals. These TFDs, however, lack the ability to resolve all components in the time–frequency plane, resulting in a loss of signal information [22].

The class of quadratic TFDs, with its selection of kernels and kernel parameters, are better able to resolve components and offer a more refined representation. Importantly, the quadratic TFDs have been successfully applied to many biomedical applications [22] and have been shown to outperform the spectrogram [23]. A critical roadblock in the more widespread application of the quadratic TFDs is the $\mathcal{O}(N^2)$ computation and computer-memory required to generate the TFD. Here, we use a separable-kernel TFD implemented by algorithms that control the oversampling, reducing the load to a more manageable $\mathcal{O}(PN)$ for computation and $\mathcal{O}(PQ)$ for memory, where $P,Q\ll N$.

Thus, we propose to design a novel CNN structure driven by a quadratic TFD of the raw EEG. This network structure is able to self-extract convolutional features based on time, frequency and time-frequency from the 2D quadratic TFD. For the first time, a large multi-centre EEG dataset of term neonates EEG is considered for investigating an automated EEG HIE-grading system.

## 2. Materials

### 2.1. EEG

Two datasets were used in this study.

#### 2.1.1. Development dataset

EEG was recorded from 54 term infants using a NicoletOne (Natus, USA) EEG system within the neonatal intensive care unit (NICU) of Cork University Maternity Hospital, Cork, Ireland. This study was approved from the Clinical Ethics Committee of the Cork Teaching Hospital with written and informed parental consent obtained before EEG recording. Detailed inclusion criteria of the cohort can be found in Korotchikova *et al* [24].

The EEG recording was initiated within 6 h of birth and continued for up to 72 h to monitor the evolution of the developing encephalopathy with seizure surveillance. The EEG recording used 9 active electrodes T4, T3, O1, O2, F4, F3, C4, C3, and Cz as standard protocol in the NICU. Our analysis used an 8-channel bipolar montage derived from these electrodes as F4-C4, C4-O2, F3-C3, C3-O1, T4-C4, C4-Cz, Cz-C3 and C3-T3.

**Figure 1.**
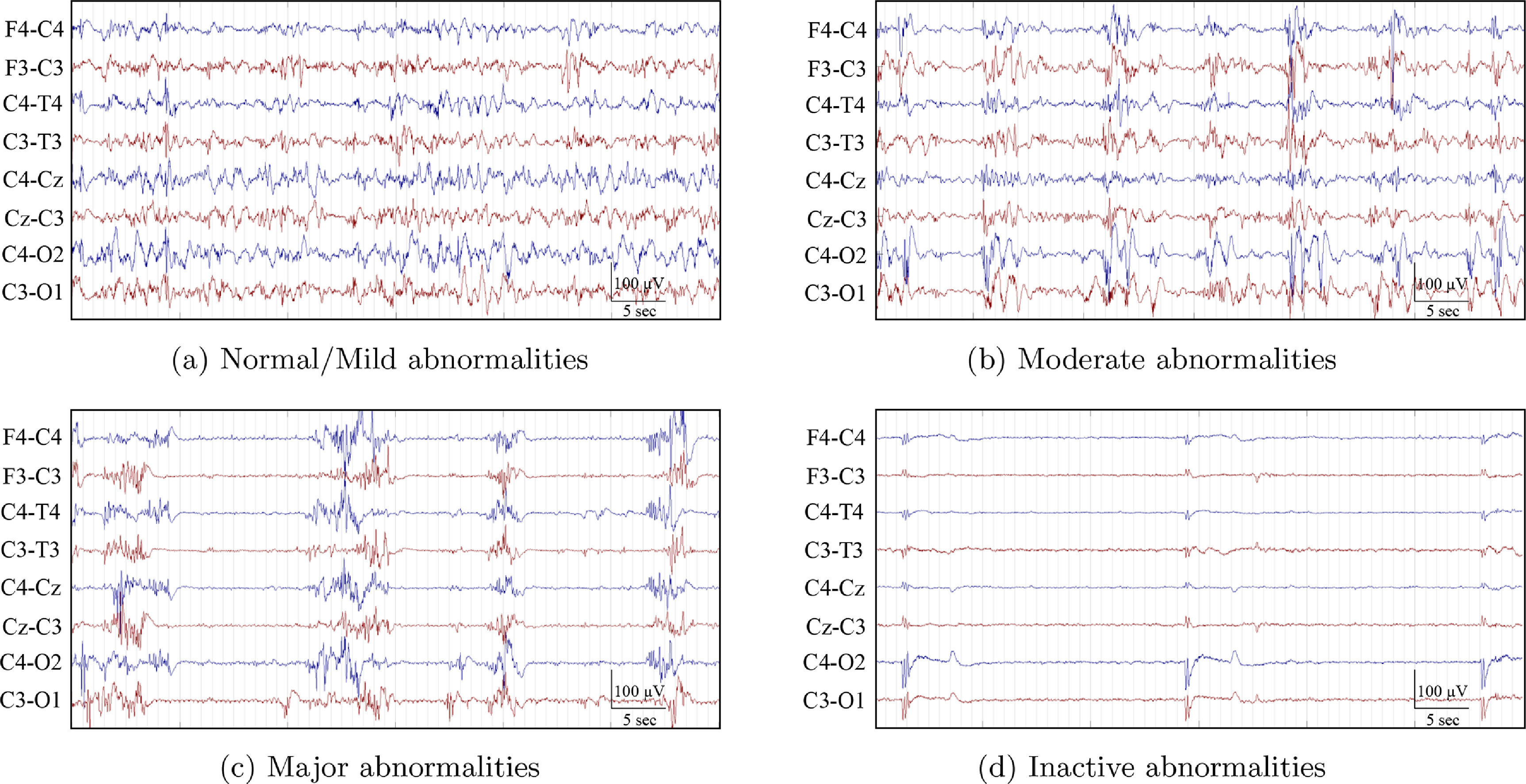
EEG examples for each of the four EEG HIE-grades.

One EEG epoch, approximately 1 h in duration, was pruned from the continuous EEG for each infant. Epochs were free of seizures and major artefacts when possible [24]. The EEG epochs were reviewed independently by 2 EEG experts and graded according to the system defined by Murray *et al* [6], summarised in table 1. Each of the 1 h epochs were assigned one of four grades corresponding to normal/mild abnormalities (grade 1), moderate abnormalities (grade 2), major abnormalities (grade 3), and inactive (grade 4). Normal EEGs (grade 0) were grouped together with normal/mild abnormalities (grade 1) during grading. Example EEGs from each grade are shown in figure 1. In the case when two different grades were assigned to the same EEG epoch, the EEGs were subsequently reviewed by both reviewers and consensus on the EEG grade was reached. The inter-observer agreement between both reviewers was high (Cohen’s kappa *κ* = 0.87).

**Table 1.** Visual interpretation of background EEG activity in hypoxic–ischemic encephalopathy defined by Murray *et al* [6].

Grade	Findings	Description of EEG
0	Normal EEG findings	Continuous background pattern with normal physiologic features such as anterior slow waves
1	Normal/mild abnormalities	Continuous background pattern with slightly abnormal activity (e.g., mild asymmetry, mild voltage depression, or poorly defined SWC)
2	Moderate abnormalities	Discontinuous activity with IBI of $\lt$10 s, no clear SWC, or clear asynchrony
3	Major abnormalities	Discontinuous activity with IBI of 10–60 s, severe attenuation of background patterns, or no SWC
4	Inactive EEG abnormalities	Background activity of ${\lt}10\ \mu$V or severe discontinuity with IBI of $\gt$60 s

Abbreviations: IBI, interburst interval; SWC, sleep–wake cycling.

This development dataset consists of 54 EEG epochs, one per infant. The mean epoch duration was 70 min (interquartile range IQR: 68–72 min). Of the 54 epochs, 22 were classified as grade 1, 14 were classified as grade 2, 12 were classified as grade 3, and 6 were classified as grade 4.

#### 2.1.2. Validation dataset

EEG was recorded from term infants from 6 NICUs across 4 European countries (Ireland, The Netherlands, Sweden, and The United Kingdom) as part of the Algorithm for Neonatal Seizure Recognition (ANSeR) study [25, 26]. All the sites had obtained approved written and informed consent before EEG recording. The EEGs were recorded either using the NicoletOne ICU Monitor (Natus, WI, USA), Xltek EEG system (Natus), or the Neurofax EEG-1200 (Nihon Kohden, Tokyo, Japan). EEG monitoring was commenced shortly after birth and was continued for up to 48 h. The multichannel EEGs were recorded with active electrodes located at F3, F4, C3, C4, T3, T4, O1, O2 (or P3 and P4) and Cz as a standard NICU protocol. We used an 8-channel bipolar montage derived from these electrodes as F4-C4, C4-O2 (or C4-P4), F3-C3, C3-O1 (or C3-P3), T4-C4, C4-Cz, Cz-C3 and C3-T3.

One-hour epochs of EEG were pruned from each neonate’s EEG recording, at 6, 12, 24, 36 and 48 h post-natal age when possible. The EEG epochs were reviewed by a clinical physiologist (S R Mathieson) with expertise in neonatal EEG. Each 1 h EEG epoch was graded according to the system proposed in Murray *et al* [6], and was assigned one of 5 grades corresponding to normal EEG findings (grade 0), normal/mild abnormalities (grade 1), moderate abnormalities (grade 2), major abnormalities (grade 3) and inactive (grade 4). As the development dataset used in this study were graded for only four grades, we combined grade 0 and 1 to compress the 5 grades to 4 for this validation set.

The ANSeR study dataset included a total of 214 neonates with valid EEGs [25]. We only included neonates with a clinical HIE diagnosis. Within this set of 141 neonates, 9 infants were excluded because of a combined diagnosis and 41 were keep out as a future validation set for further algorithmic development. The remaining 91 neonates, providing a total of 338 1 h 8-channel EEG epochs were used as the validation dataset. Of these 338 epochs, 188 were classified as grade 1, 81 were classified as grade 2, 38 were classified as grade 3, and 31 were classified as grade 4. More details about the cohort can be found in Rennie *et al* [25].

### 2.2. Baseline systems

Four algorithms from our group that were previously developed and tested on the development dataset in section 2.1 are compared with the proposed algorithm. This helps to have a fair comparison between these methods and the one proposed in this paper. The methods are summarised below.

#### 2.2.1. Quadratic TFD features (TFDfeat)

This method uses non-stationary features of a quadratic TFD (separable-kernel TFD), in addition to generic quantitative EEG features, which were combined with a linear-discriminate classifier [7]. First, the amplitude modulation (AM) and instantaneous frequency (IF) are extracted from the TFD. Second, statistical measures of the distribution of AM and IF are used to summarise the non-stationary amplitude and frequency modulation of the EEG. Next, these features are coupled with spectral power and combined in the classifier on short-term EEG segments (64 s). Majority voting is then used to convert short-term decisions into one grade for the 1 h epoch. This was the first method developed on the developmental dataset.

#### 2.2.2. IBI features (IBIfeat)

This method uses an IBI detection algorithm to quantify the duration and persistence of IBIs in the EEG [11]. Duration of the intervals is an important component in the grading scheme—see table 1. Although the inter-burst detection algorithm was previously trained on preterm infant EEG, we found it worked well to detect the inter-burst intervals in the EEG of HIE term infants. Features of IBIs, extracted from 10 min segments, were combined using a multi-layer perceptron classifier. Majority voting over the hour determined the grade. Although only 2 features were used, this method had similar performance to the TFDfeat method [7].

#### 2.2.3. One-dimensional CNN (CNN1d)

This method uses a 1-dimension CNN on the raw EEG from each 5 min EEG segment [13]. The features, extracted by the convolutional layers, are classified by two fully connected hidden layers followed by a softmax layer. The output probabilities across channels are combined with two-step voting to determine the grade over each 1 h EEG epoch. This method, which learns hierarchical representations from the raw EEG, outperforms the traditional hand-crafted features approach of the IBIfeat and TFDfeat methods [7, 11].

#### 2.2.4. GMM supervectors (GSVfeat)

This method combines a generative model with a discriminative classifier [10]. First, a set of 55 short-term features in frequency, time, and information theory domains are extracted from 8 s EEG segments with 50% overlap. Second, the sequence of short-term feature vectors are compiled over an 80 s segment to generate a Gaussian mixture model (GMM). All the data is then used to generate a universal background model. This background model is then compared to each 80 s segment under analysis and parameters of similarity between the 2 are extracted. These model-distance measures, known as supervectors, are then classified using a support vector machine (SVM). This method also employs different post-processing strategies to combine the decisions over the 1 h epoch and across the 8 channels. The method has the best performance to date for the developmental dataset (section 2.1) and therefore will be compared with the proposed system on the validation dataset.

#### 2.2.5. Other state-of-the-art methods

Methods developed on different datasets, and therefore not included as a direct comparison to the proposed method, includes a tensor-decomposition method by Matić *et al* [8], an inter-burst interval detector method also by Matić *et al* [9], and a features-based approach by Guo *et al* [12].

## 3. Methods

The proposed grading system consists of a CNN driven by a quadratic TFD of a segment of single-channel EEG. The CNN has three parallel layers that first convolves the TFD in the time, frequency, and time–frequency directions. These independent layers are followed by multiple convolutional layers (with a nonlinear operator) and pooling layers as usually defined in CNNs. Classification is performed by two final fully-connected layers of neurons.

### 3.1. EEG time–frequency transformation

All bipolar EEG channels are low-pass filtered to 30 Hz frequency using a finite-impulse response (FIR) filter with zero-phase. The filter was designed using a Hamming window of length 4001 samples. The EEG was then downsampled from the original sampling frequency to 64 Hz.

A quadratic time–frequency distribution (TFD) is used to transform the EEG signal *x*(*t*) to the time–frequency domain *ρ*(*t*, *f*). The Wigner–Ville distribution (WVD) is the base distribution for the class of quadratic TFDs [22]. It is defined as,

1 \begin{equation*} W(t,f) = \int_{-\infty}^{\infty} z(t+\tfrac{\tau}{2}) z^{*}(t-\tfrac{\tau}{2}) \textrm{e}^{-\textrm{j}2 \tau f} \textrm{d}\tau, \end{equation*}

where *z*(*t*) is the analytic associate of *x*(*t*) and z*(*t*) represents the complex conjugate of *z*(*t*) [27]. A two-dimensional smoothing kernel *γ*(*t*, *f*) is convolved in time and frequency with the WVD,

2 \begin{equation*} \rho(t,f) = W(t,f) \underset{t}{*} \underset{f}{*}\; \gamma(t,f), \end{equation*}

to produce the class of quadratic TFDs. We use a separable kernel of the form *γ*(*t*, *f*) = *G*(*t*)*H*(*f*) to independently control smoothing in the time- and frequency directions [22, 28, 29].

The kernel function *G*(*t*)*H*(*f*) is defined as a two-dimensional low-pass filter in the Doppler–lag domain (ambiguity domain) as *g*(*ν*)*h*(*τ*) [22]. Although the main purpose of the kernel is to suppress cross- and inner-terms from the WVD, it also creates a level of abstraction for the TFD and therefore the signal. The more smoothing in (2), the more general the TFD becomes which, in turn, represents a larger class of signals [28].

The TFD is evaluated on a 5 min segment of an EEG epoch. This duration was based on previous analysis using the same training data set [13]. For the discrete implementation of (2) we use a fast and memory-efficient algorithm that avoids oversampling in the time- and frequency-directions [28, 30]. We select short-duration Hann windows for both the Doppler *g*(*ν*) and lag *h*(*τ*) kernels. Shorter-duration windows provide sufficient abstraction of the EEG signal with the added benefit of faster computation and reduced memory requirements. The generated TFD is a 256 × 128 matrix, which requires a small fraction (${\lt}0.01$%) of memory comparative to the full (over-sampled) TFD of dimension 38 400 × 19 200. The length of the Doppler window is set to 127-samples and the lag window is set to 63-samples as approximately half of the kernel window size (256 × 128) used for training on the development dataset. For testing validation dataset performance, a grid search is performed in a nested 5-fold cross validation on first training iteration of the development dataset to evaluate the best length of the Doppler and lag windows (details in section 4.2).

**Figure 2.**
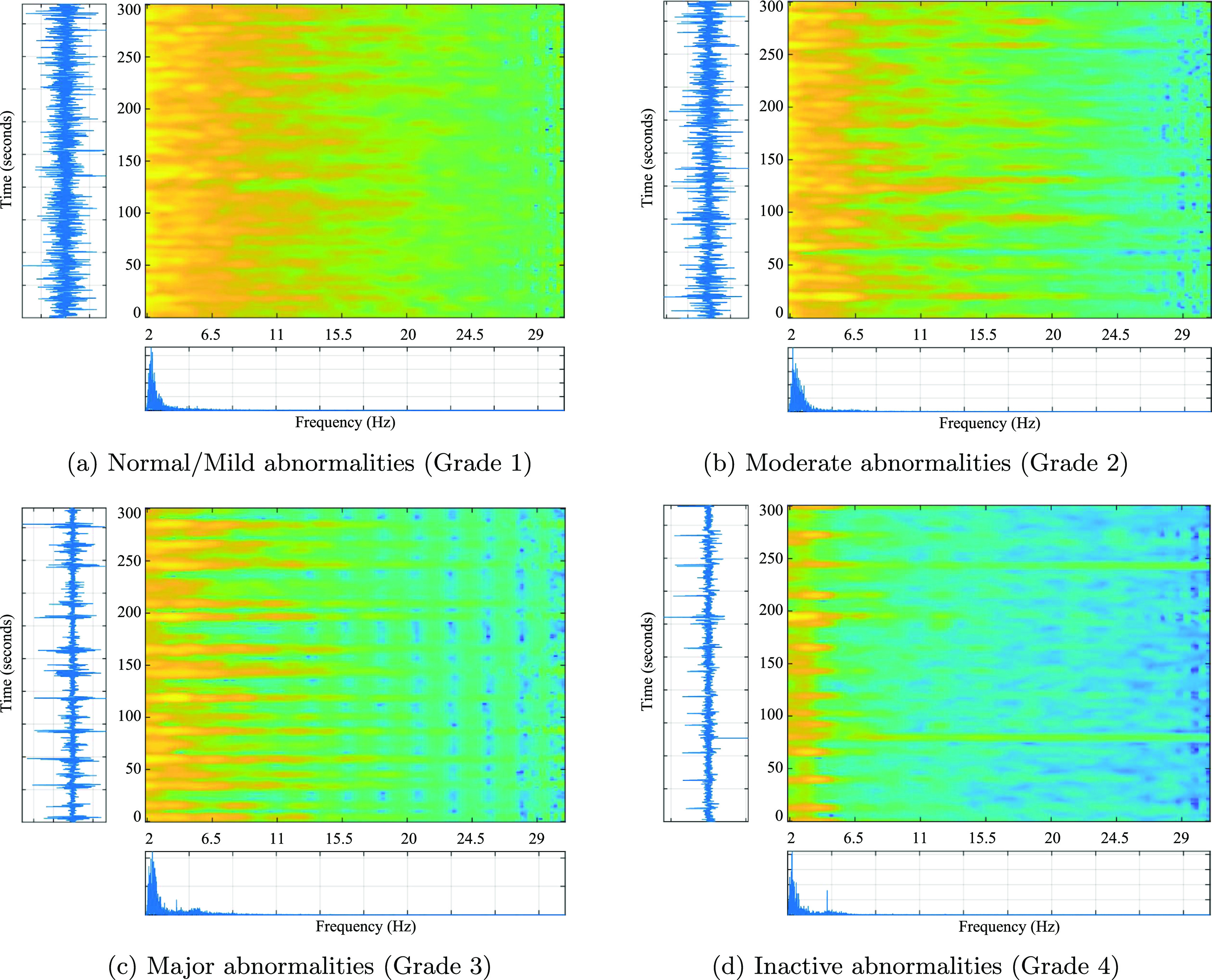
Examples of the time–frequency distributions for 1 channel of a 5 min EEG segment for each of the 4 HIE grades.

Before generating the TFD, a simple pre-whitening filter is used to flatten the spectrum and emphasise the higher-frequency components of the TFD. This is implemented using the derivative of signal, estimated using the forward-finite difference method [28]. The low frequency components at 0–2 Hz and high frequency components at 30–32 Hz are removed from the TFD to reduce the wrap-around effects, thus producing a final 256 × 112 matrix. Lastly, the log of the absolute TFD matrix is used as input to the CNN. Figure 2 shows TFD examples of 5 min EEG segments with different HIE grades.

### 3.2. CNN model

**Figure 3.**
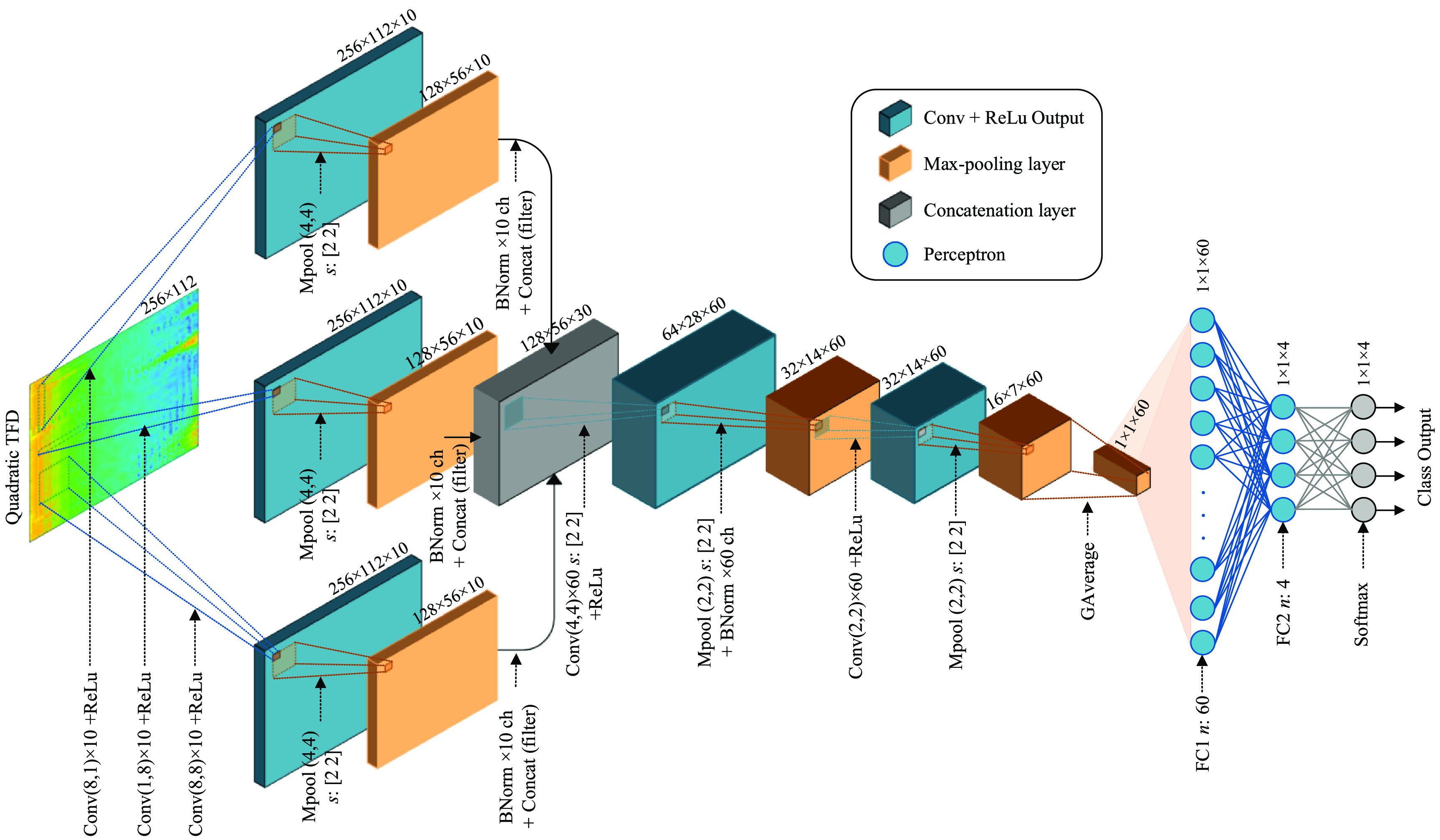
Structure of the proposed CNN model. The input is a 2-dimensional TFD matrix of a 5 min EEG segment from one channel and the output is 4 probabilities corresponding to the target classes. Abbreviations: Conv, convolutional layer; ReLu, rectified linear unit; MPool, pooling by maximum operator; BNorm, batch normalisation; GAverage, global averaging; FC, fully connected layer; *s*, stride; *n*, neuron.

The proposed CNN architecture comprises of convolutional, rectifying, pooling, and normalisation layers for feature extraction; with classification carried out by two fully-connected layers with a softmax layer. The convolution layer performs a two-dimensional filter-bank operation using finite-impulse response filters $\mathbf{Y}_k = \mathbf{F}_k \ast_t\ast_f \mathbf{X}$ for the *k*th filter with $\mathbf{X}$ as the input matrix and *as the convolution operation without padding. The matrix $\mathbf{F}_k$, which contains the filter coefficients including a bias term, are learnt during training. This layer is followed by a rectified linear unit (ReLu) non-linearity, which maintains the input positive values and replaces the negative values by zero as $\mathbf{{max}}(0, x)$. Next, a pooling layer after each successive convolutional–ReLu layers is used to extract features. Also, it reduces the amount of parameters to train and helps to limit overfitting. The pooling layer takes the maximum value over a prescribed window for downsampling the volume of the previous layer. Batch-normalisation layers are used to normalise the extracted feature maps. For classification, two fully connected hidden layers, equivalent to the multi-layer perceptron, are included. Finally, a softmax layer is used to exponentially normalise the network outputs and represent them as probabilities corresponding to the target classes. The detailed layer structure for proposed CNN architecture is illustrated in figure 3.

The TFD (256 × 112) of a 5 min EEG segment is input to a structure with three parallel convolutional layers. The first convolutional layer evaluates a feature map in the time domain by convolving 10 different (8 × 1) filters, $\rho(t,f) \ast_t \mathbf{F}_k$. The output of the convolutional layer is passed through the ReLu layer. Next, a max-pooling layer is included, which takes the maximum value from a sliding window segment (size 4 × 4), with [2 × 2]-stride downsampling. This results in a dimension reduction from (256 × 112) to (128 × 56) sample-points across 10-feature maps. Further, the batch normalisation layer is included to normalise the evaluated feature map across each of the 10-filters. The second convolution layer evaluates a feature map in the frequency domain by convolving 10 (1 × 8) filters, $\rho(t,f) \ast_f \mathbf{F}_k$ without padding. Finally, in the third convolution layer structure, the feature map is evaluated in time–frequency by convolving 10 (8 × 8) filters, $\rho(t,f) \ast_t\ast_f \mathbf{F}_k$ with no padding. Both convolution structures are followed by rectification, pooling and normalisation process with similar configuration as described in the first convolution structure.

The evaluated time, frequency, time–frequency feature maps from the convolution structures are integrated using a concatenation layer of dimension (128 × 56 × 30). Further, a convolution layer is used to evaluate a feature map by convolving in (4 × 4) dimension across 60-filters with [2 × 2]-stride downsampling resulting in a (64 × 28 × 60) feature map. This layer is followed by another ReLu layer. Next, the maxpooling layer is used with a (2 × 2) sliding window across 60-filters, using a [2 × 2]-stride downsampling. It is followed by a batch-normalisation layer across 60-channels resulting in a (32 × 14 × 60) feature map. At the final convolution stage, a (2 × 2) convolution evaluates a feature map across 60-filters, followed by a Relu layer. The maximum pooling operation is evaluated on a (2 × 2) sliding window, downsampling at [2 × 2]-stride. The final layer of this process is a global-average layer which produces only 1 sample point for all 60 feature maps. The classification is then performed by two fully connected hidden layers with 60 and 4 neurons in each layer. Finally, the softmax layer provides the output class probabilities of size (1 × 4).

The integrated regularisation within the CNN shared weights and sparse connectivity results in fewer trainable parameters and therefore limits overfitting. Hence, no early stopping criteria was found necessary in this study. The loss function used was categorical cross-entropy. Stochastic gradient descent was used with an initial learning rate of 0.01, this was reduced by 20% every 5 iterations. Nesterov momentum was set to 0.9. A batch size of 128 was used for training and validation.

### 3.3. Post-processing

**Figure 4.**
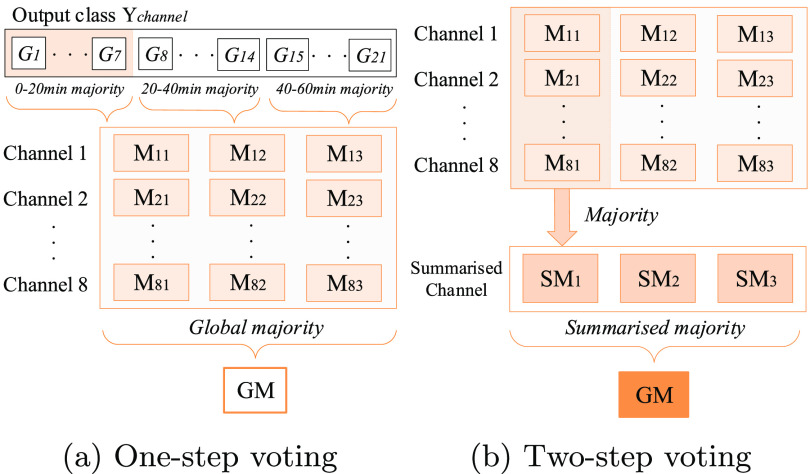
Post-processing strategies of assigning an overall grade for a 1 h EEG epoch. *G*_*m*_ represents the grade from the CNN output for each 5 min segment. $\textrm{M}_{\textrm{cn}}$ represent majority vote of *G*_*m*_ over *m* = 1, 2, …, 7 for channel *c*. SM_*n*_ is the majority vote of $\textrm{M}_{\textrm{cn}}$ over channels *c* = 1, 2, …, 8. GM is the global majority vote evaluated from either matrix $\textrm{M}_{\textrm{cn}}$ or vector SM_*n*_ over *n* = 1, 2, 3.

The CNN generates an output class label for each processed 5 min EEG segment from each channel. We evaluate 2 different post-processing strategies to estimate the EEG HIE-grade for the 1 h epoch. Figure 4 illustrates the process for the 2 strategies.

**Figure 5.**
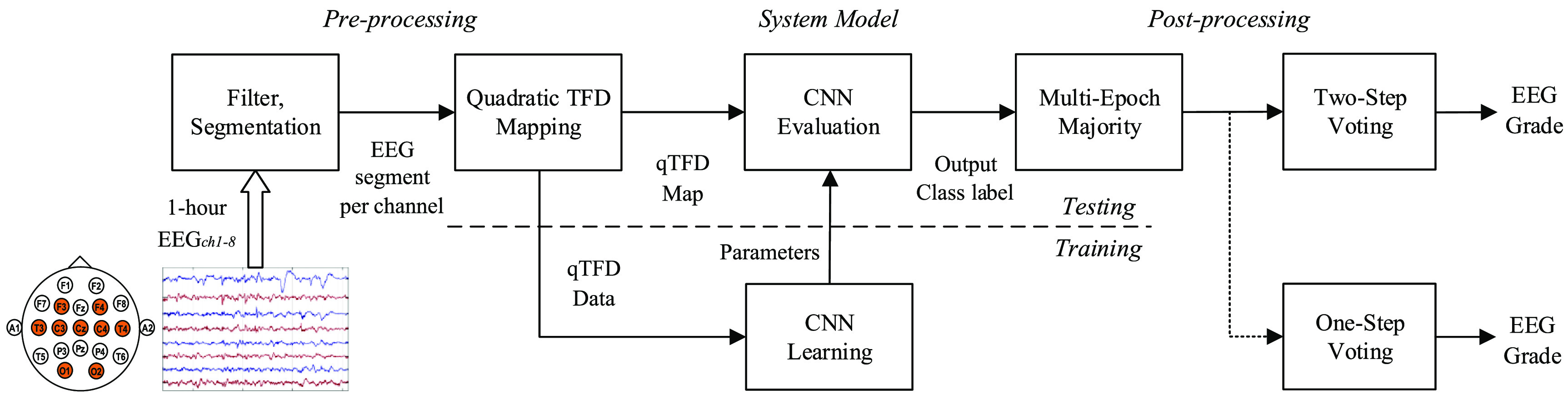
Proposed EEG HIE-grading system for term infant. Multi-epoch majority evaluates a majority grade from 20 min. Abbreviations: TFD, time–frequency distribution; CNN, convolutional neural network.

The majority grade from 7 EEG segments (5 min segments with 50% overlap) is evaluated over each 20 min period, resulting in 3 predictions per channel for the 1 h epoch. This is repeated for all eight channels, resulting in a 8 × 3 matrix $M_{\textrm{cn}}$. The overall grade is calculated by either the majority vote of this matrix $M_{\textrm{cn}}$ (one-step voting) or by first taking the majority votes across channels, each column *c* of the matrix $M_{\textrm{cn}}$ for *n* = 1, 2, 3, and then across the summarised row (two-step voting). The overall structure of the proposed system is shown in figure 5.

For training and testing, a leave-one-subject-out (LOSO) cross-validation method is used on the development dataset (section 2.1.1). In training, for a 1 h epoch, a TFD matrix is generated for each 5 min epoch (with 50% overlap) for each channel. All segments within this hour are assigned the same label. There will therefore be 21 separate training instances within each hour-long epoch, each with a different TFD as input. Based on the 54 infants in the development dataset, system performance was calculated by training the CNN model with the EEG epochs from 53 infants and testing on the one left out. This was repeated until all 54 infant’s EEG epochs were tested to determine the overall classification accuracy of system. Next, the trained model of the 1st LOSO iteration from development dataset is tested on the unseen 338 EEG epochs from the validation set (section 2.1.2). The GSVfeat system from section 2.2.4 is also tested on the validation dataset using the trained model from 1st the LOSO iteration.

### 3.4. High-amplitude artefacts

High-amplitude artefacts in the EEG are often produced by physical movement of the electrode leads. Movement or handling of the infant can cause the wires connected to the electrodes to move. Those infants receiving hypothermia therapy will be sedated and thus less likely to move or to be moved for feeding, burping, or parental holding, though an initial period where IV lines and monitoring sensors are attached may involve frequent handling. Infants with moderate or severe grades of HIE will be actively cooled and therefore likely to have less high-amplitude artefact overall compared to those infants with normal or mild EEG grades. To test this hypothesis, we quantify the percentage of high-amplitude artefact and compare with the EEG grades and with the algorithm’s estimated grades. The proposed algorithm was trained on raw EEG, without an artefact removal stage. However the presence of high-amplitude artefacts in each 5 min epoch is quantified and related to classification performance.

We modify an existing approach to detect the high-amplitude artefact in neonatal EEG [31]. Each channel of EEG is bandpass filtered from 0.5 to 10 Hz using a 5th order Butterworth infinite-impulse response (IIR) filter. The system has 2 parts. First, the system detects very high-amplitude activity. If any point of the envelope of the signal, estimated from the analytic signal, exceeds a threshold of 300 *µ*V, this peak with a time-collar of 10 s before and 10 s after this peak is labelled as artefact [31]. As this is very high-amplitude activity, it is likely to be present on more than one channel. Therefore all EEG channels within this hemisphere and at this time point are also labelled as artefact. The process iterates for all channels on the both the left and then right hemispheres. (There are no mid-line channels in our analysis.) Second, a channel-by-channel process detects remaining high-amplitude activity with an EEG envelope greater than 100 *µ*V (and ≤300 *µ*V as this is applied after the first part). This time a 3 s time-collar is applied to the peak and then labelled artefact for that channel only.

The total percentage of artefact (%artefact) per epoch is estimated. To test the difference in %artefact for the 4 EEG grades, an omnibus test (Kruskal–Wallis) is applied and, if significant ($P\lt0.05$), is followed by pair-wise comparisons using Tukey’s honest significant difference test. In addition, the %artefact is correlated with the grades using the Spearman correlation coefficient with a 95% confidence interval (CI). This CI is generated using a bootstrap process. Next, the %artefact is averaged for each entry in the confusion matrix between the proposed algorithm and the EEG grades. The average %artefact for correct classifications (diagonal entries of the confusion matrix) is compared with the average %artefact for the incorrect classifications (the off-diagonals of the confusion matrix). To determine if the difference between the %artefact for correct and incorrect classifications is significant, the CI of the paired difference is generated using a bootstrap procedure with random sampling of the infant EEGs, not the epochs. All bootstrap processes use 1000 iterations to generate the distributions.

## 4. Results

**Figure 6.**
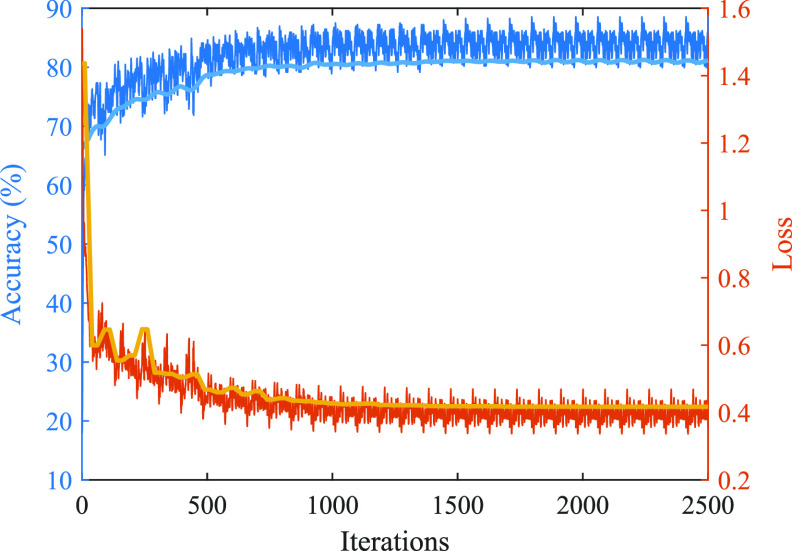
CNN training–validation accuracy and loss over training iterations. Dark blue and orange shade represents training results, light blue and yellow shade represent validation results.

To evaluate the CNN training–validation performance, the first LOSO training iteration of development dataset is split randomly in 90:10 as training–validation set. The network is trained based on the 1st LOSO iteration and training–validation results over learning iterations are shown in the training curve in figure 6. It converges to a stable validation accuracy after approximately 2000 learning iterations.

### 4.1. Training results

Within the LOSO cross-validation on the development dataset, the effect of the different post-processing techniques on system performance using the 5 min EEG segments is illustrated in table 2. The *CNN output* in table 2 is evaluated based on majority vote from a total of 184 segments (23 per channel) from all 8 channels within an 1 h EEG epoch. The one-step voting summarises over time (20 min) and channels simultaneously; the two-step voting first summaries over channels and then over time. The two-step voting shows highest system performance and thus was used to analyse the proposed system performance in the final system pipeline.

**Table 2.** Comparison of system performance on development dataset based on different post processing techniques.

	CNN output	One-step voting	Two-step voting
Accuracy	83.3%	87.0%	88.9%
kappa	0.77	0.81	0.84

The confusion matrix of the testing results for the proposed system using two-step voting is presented in table 3. The green shaded cells along the main diagonal show the correctly classified segments and the pink shaded cells indicate the groups with highest number of falsely classified EEG epochs. It shows that 48/54 (88.9%) EEG epochs were correctly classified. In addition, no incorrect classifications were more than 1 grade away from the correct class.

**Table 3.** Confusion matrix based on the convolutional neural network output with two-step voting.

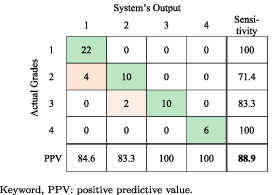

The performance of existing systems, which were developed on the same EEG dataset, are compared with our proposed system in table 4. Of all the feature-based methods, the best performance was obtained by GSVfeat method with an accuracy of 87% and level of agreement *κ* = 0.81. The CNN1d method shows comparable accuracy of 81.5% and level of agreement *κ* = 0.73. Our proposed system, with an accuracy of 89% and *κ* = 0.84 and which operates the need for a feature extraction strategy, also has comparable accuracy to the GSVfeat method and outperforms the CNN1d approach.

**Table 4.** Comparison of the proposed with existing methods developed on the same database.

	TFDfeat [7]	GSVfeat [10]	IBIfeat [11]	CNN1d [13]	Proposed method
Accuracy	77.8%	87.0%	77.8%	81.5%	88.9%
kappa	0.68	0.81	0.68	0.73	0.84

And lastly, performance for the proposed system is compared with the 2 EEG graders in the context of inter-grader agreement. System performance is *κ* = 0.76 when using grader-1 annotations and *κ* = 0.84 when using grader-2 annotations (grader-2 annotations were similar to final consensus annotations used for training and testing). Both these measures were below inter-rater agreement between grades 1 and 2, which was *κ* = 0.87.

### 4.2. Validation results

**Figure 7.**
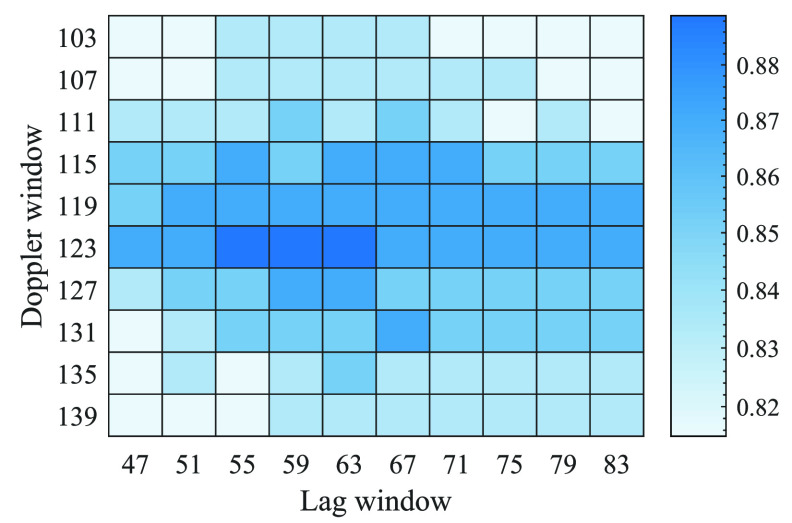
Proposed system performance as function of TFD parameters: Doppler and lag window length. Testing results using a nested 5-fold cross-validation within the 1st leave-one-subject-out iteration of the development dataset.

The best kernel parameters for the quadratic TFD parameters (length of the Doppler and lag windows) are evaluated from a grid search in a nested 5-fold cross validation on the first LOSO iteration of the development dataset. Based on this grid search (see figure 7), we set the length of Doppler and lag windows to 123- and 59-samples for the quadratic TFD. The proposed CNN model, re-trained on the first LOSO iteration of development dataset with the selected kernel quadratic TFD parameters, is tested on the unseen validation dataset.

The confusion matrix for the proposed system is presented in table 5(a) for testing on the 338 EEG epochs from 91 infants from the validation dataset. Additionally, the leading baseline system (GSVfeat) is also tested on the validation dataset, performance of which is illustrated in table 5(b).

**Table 5.** Confusion matrix for validation dataset.

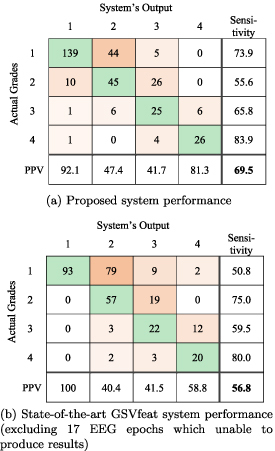

The proposed system output correctly classified 235/338 (69.5%) EEG epochs, with *κ* = 0.54. The state-of-the-art GSVfeat method correctly classified 192/338 (56.8%) EEG epochs, with *κ* = 0.39. From the 146 incorrect classifications, 17 were void because the system was unable to produce an estimate because of artefact. Table 6 compares accuracy for the 2 methods. The distribution of difference in accuracy is generated by a bootstrap procedure with resampling on the subjects (not the epochs). We find a clear separation in results: the proposed method has an increase in accuracy of 12.6% (95% CI: 7.4%–18.2%; $P\lt0.001$) over the state-of-the-art method.

**Table 6.** Difference in classification performance (accuracy, with 95% confidence intervals) between the proposed and the state-of-the-art GSVfeat system for the validation dataset.

GSVfeat system	Proposed system	Difference	*P*-value
56.8%	69.5%	12.6%	$\lt$0.001
(51.8–62.1)	(65.3–73.6)	(7.4–18.2)	

**Figure 8.**
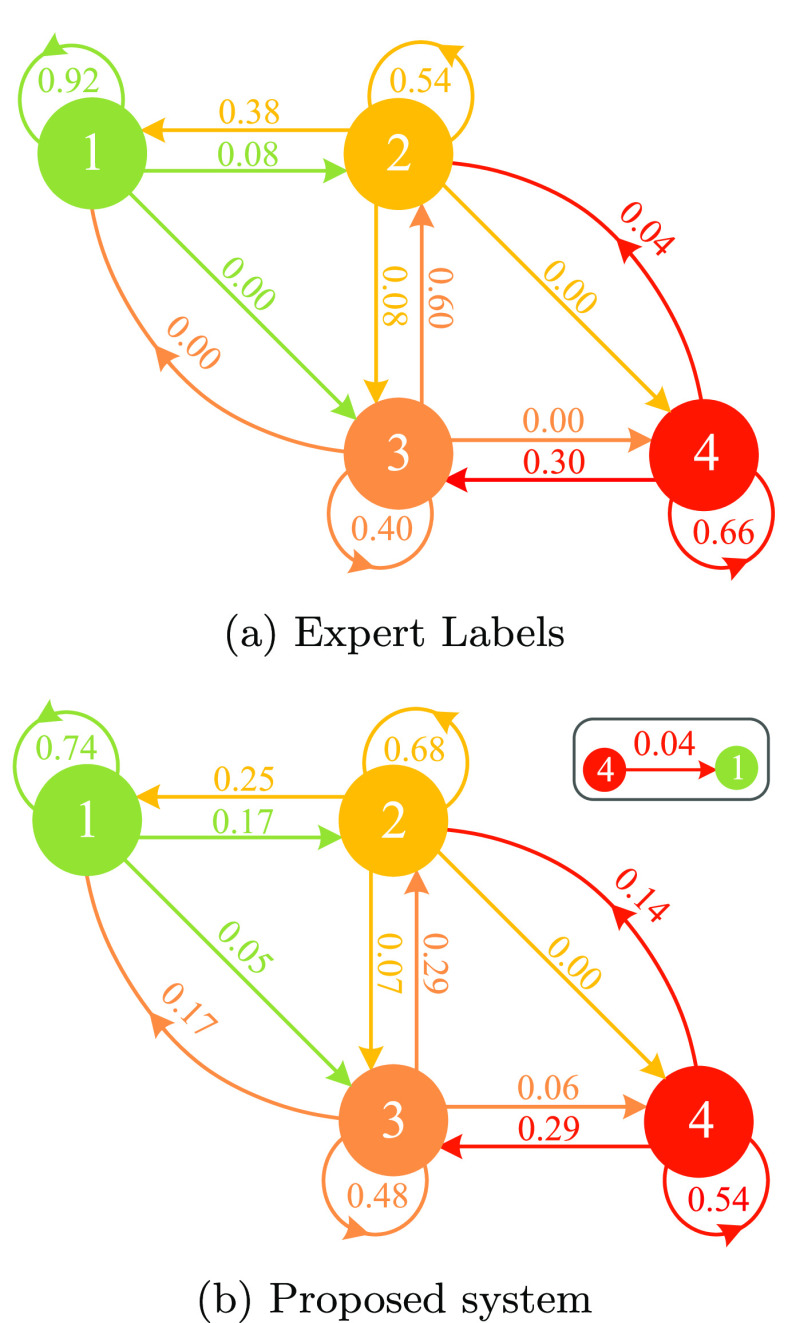
Graphs shows the transition probabilities between the EEG HIE-grades based on the experts’ labels and automated proposed systems. The values shown on each graph illustrates the probability of shifting to the state/grade.

The validation dataset contains EEG epochs extracted at specific time points, from 6 up to 48 h after birth (see section 2.1.2). The EEG grade can change over time, a consequence of clinical intervention or because of the evolving nature HIE. To model the transition of the EEG grades with time after birth (postnatal age), we apply a Markov analysis to determine transition probabilities. Postnatal age is considered as a categorical variable and therefore relative time between EEG epochs is not considered here. Figure 8 shows the 4-state transition graphs evaluated for the expert labels and the proposed system. The transition graphs have 4 nodes representing each EEG HIE-grade. Each node has 3–4 outward links, one of which represent the self-loop edges defining the probability of remaining in the same state.

The transition probability for the proposed system (figure 8(b)) was calculated from the evaluated two-step voting output for each one-hour EEG epoch. It shows weaker transition probability between grades 3 and 2 (with *P* = 0.29) compared to the experts’ transition probability (figure 8(a)) of *P* = 0.60 and an increase from 0.40 to 0.48 in probability to maintain as grade 3. Whereas, the transition probability between grade 4 and 2 shows reasonable transition as compared to experts’ label in figure 8(a). The 2-step grade transitions shows weaker probability for the proposed system versus the experts labels. Also, the proposed system has 1 non-zero probability (*P* = 0.04) for 3-step transition; the expert’s labels have none.

**Figure 9.**
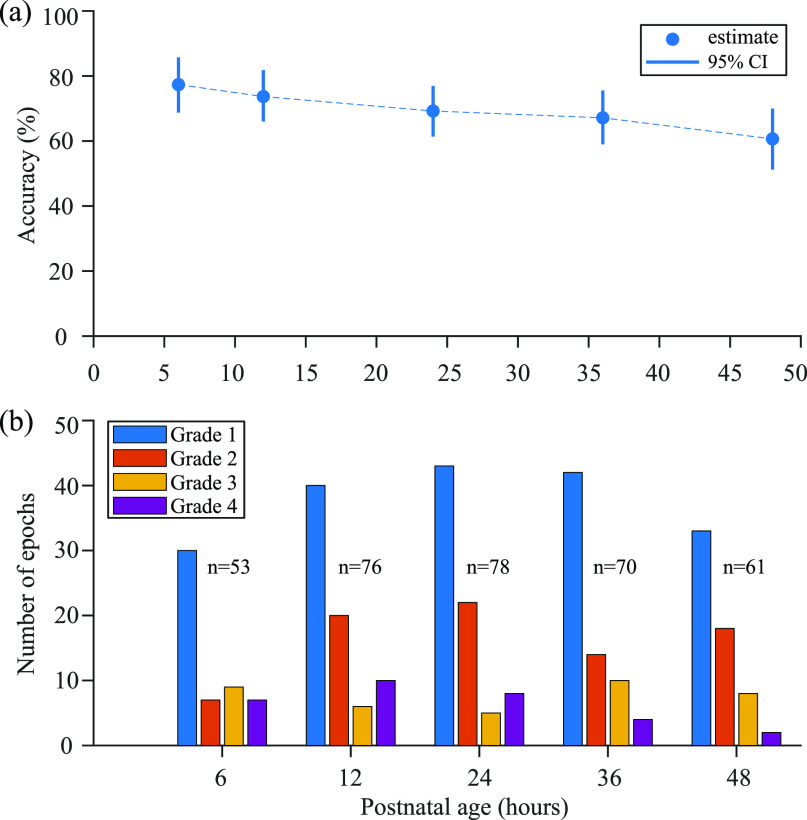
Performance for the proposed system over the different time points. (a) Accuracy with 95% confidence interval (CI) and (b) distribution of grades in the data set vary for the different time points; *n* is the total number of epochs and therefore neonates at each time point.

The proposed system performance is also evaluated separately at the different time-points, as shown in figure 9. The system shows a slight decrease in accuracy over time. This could be a consequence of the unequal distribution of grades at the different time points in figure 9(b).

In many clinical settings amplitude-integrated EEG (aEEG) monitors are used which provide a highly processed and condensed representation of the EEG. Such monitors use a limited number of electrodes, and typically display two channels of aEEG. Our system was trained with eight channels of EEG using a channel independent training strategy. To show the potential application of this method for use in an aEEG machine, the trained system was tested over the validation dataset using only two channels (F3-C3 and F4-C4). One-step majority voting (over the 1 h epoch) was used. Performance comparing the 8- versus 2-channels testing is summarised in table 7.

**Table 7.** Testing performance for the reduced versus full montage (accuracy, with 95% confidence intervals) over the validation dataset.

2-Channel system	8-Channel system	Difference	*P*-value
69.5%	69.5%	0.0%	0.880
(65.5%–70.0%)	(65.3%–73.6%)	(−3.4%–4.0%)	

The reduced 2-channel EEG system correctly classified 235/338 (69.5%) EEG epochs, *κ* = 0.54 for the validation dataset, similar to the standard 8-channel EEG system. Although these summary measures (accuracy and kappa) are exactly equal, their confusion matrices differ.

**Figure 10.**
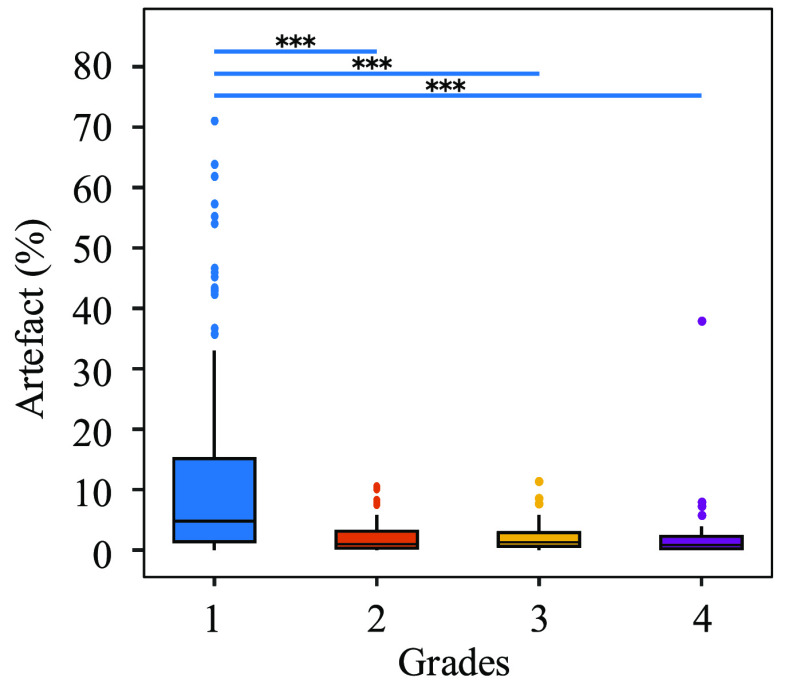
Presence of high-amplitude artefact for the 4 EEG grades in validation dataset. Tukey’s honest significance tests shows differences between grade 1 and grades 2 to 4 (*** represents *P* < 0.001).

The impact of artefact on different grades within the validation dataset is evaluated based on high amplitude artefact detection strategy described in section 3.4. The artefact detection procedure labelled a median of 2.5% (IQR: 0.7%–7.6%; range: 0.0%–71.3%) of the EEG as high-amplitude artefact in the validation data set. There was significantly more artefact ($P\lt0.001$; Tukey’s honesty difference test) in grade 1 compared with grades 2–4; see figure 10. There was a significant correlation between %artefact and grades, with *r* =−0.40 (95% CI: −0.49 to −0.31; $P\lt0.001$). The average %artefact for the correct classifications was 6.24% versus 4.68% for incorrect classifications. This difference was not significant: the paired difference was 0.48% (95% CI: −3.67 to 3.18%; *P* = 0.784).

## 5. Discussion

The main contribution of this study is a novel approach of employing quadratic TFDs with a deep CNN that inherently extracts and optimises features to classify four EEG HIE-grades. The use of the quadratic TFD provides a layer of signal abstraction to better represent the multiple and complex waveforms associated with different grades of EEG. We show that the TFD–CNN outperforms that of a CNN operating on the raw one-dimensional signal [13]. This method shows significant improvement in both development and validation datasets compared to the current state-of-the-art hand-crafted feature based systems [7, 10, 11].

Other state-of-the-art methods, developed and tested on different datasets, reported strong performance metrics. The system developed by Matić *et al* [8] reported an accuracy of 89% for classifying three EEG-HIE grades on a dataset of 34 neonates. The time and frequency-based feature system by Guo *et al* [12] reported an accuracy of 79.5% for classifying 3 EEG-HIE grades from 64 neonates. The IBI feature-based approach by Matić *et al* [9], achieved a 4 EEG-HIE grades classification accuracy of 95% on a dataset of 38 neonates. It is difficult to directly compare these methods with our proposed method, however, as the testing datasets and classification grades differed (3 instead of 4 grades for some methods). The inability of direct and fair comparison impedes scientific progress. This limitation underscores the need for a publicly available data set or a competition-based platform to compare and rank methods using the same validation data and grading scheme.

The proposed system was trained and tested on the development dataset of 54 1 h epochs from 54 neonates. This enabled a fair comparison with our group’s existing methods: TFDfeat [7], IBIfeat [11], CNN1d [13], and GSVfeat [10]. The larger data set, of 338 1 h epochs from 91 neonates, was left for validation only. This unseen data set allows for a better estimate of the generalisation error as there is a natural tendency, even unconsciously, to tune algorithms even within the theoretical unbiased leave-one-out cross-validation process. The leading GSVfeat system [10] for 4-EEG HIE-grades classification was considered as state-of-the-art system and tested on the same dataset for direct comparison. The baseline GSVfeat system reported in table 5(b) shows an overall accuracy of 56.8% for classifying 4-EEG HIE-grades compared to our proposed system with 69.5% accuracy.

**Figure 11.**
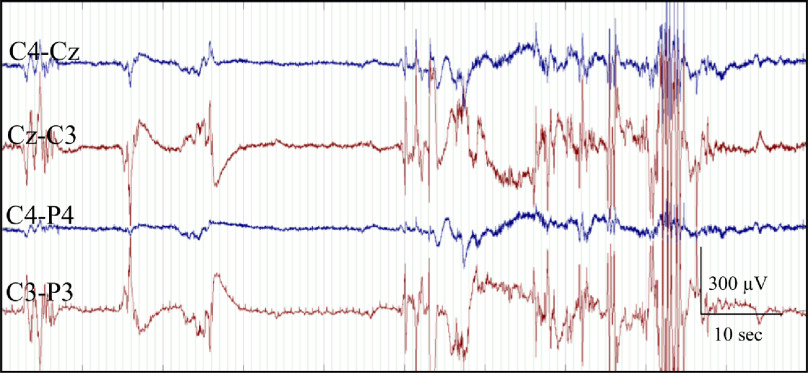
Representation of a period of artefact presence in grade 4 EEG misclassified as grade 1 in validation dataset.

As illustrated in table 5(a), the misclassification is usually only 1 grade away, with limited exceptions; one such exception is a grade 4 classified as a grade 1. On inspection, we found that there was considerable noise activity in the form of spikes and sharp waves, particularly over the left hemisphere (figure 11). During the initial EEG review, the neurophysiologist ignored these artefacts and based the grading decision on the extensive low-amplitude background present throughout the recording.

Since our proposed system determines the grade based on majority output instances over 5 min duration segments across all channels, the algorithm’s decision was likely influenced by the artefact rather than the low-amplitude activity. Our system did not include an artefact detection or rejection module. This suited our development process, which included a relatively artefact-free data set. The validation dataset however does contain more artefacts than the development set. These could explain, in part, the drop in performance from the development to the validation dataset.

**Figure 12.**
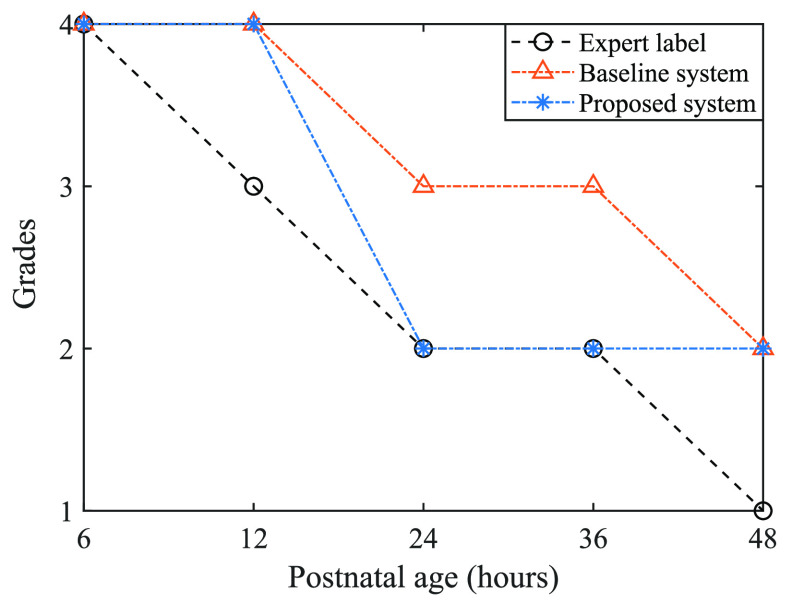
Example of EEG grade transition with progressed post-natal age (time after birth) for one newborn.

The proposed system shows gradual decline in performance for the validation dataset after the 6 h time-points, as shown in figure 9. HIE being an evolving injury, the transition of grade can improve in some cases and worsen in others. As per the expert-labels transition shown in figure 8, the strongest grade transitions are self transitions (i.e. no transition), particularly with grade 1. The 2nd strongest are with a decrease in grade and only very weak transitions up a grade ($P\lt0.1$), and no transitions (other than 4 to 2 with *P* = 0.04) between 2 grades. Thus, such transition could results in misclassification when an hour-long EEG epoch is close to the border between two grades. An example of grade transition in progressive time-points and evaluated by proposed and baseline system (GSVfeat) is shown in figure 12.

Here, the grade progresses from 4 to 1 over the transition from 6 to 48 h. Our proposed system follows this trend with 3/5 correct classifications. The baseline system similarly follows this trend but only with 1/5 correct classifications. The misclassifications for the proposed system is incorrectly holding the previous time-point grade. These grades may be more difficult to classify as the injury and matching EEG is evolving continuously, even within the 1 h epochs. The post-processing component of our proposed system produces a discrete label only without any form of certainty in this decision. There may be clinical value in including a certainty metric in addition to the decisive labelled output. This could be constructed in a similar fashion to that described in Ahmed *et al* [10], by first averaging the probabilities from the CNN of the four individual grades across the channels and then combining them to form a single likelihood measure. A confidence interval on this measure could be constructed from the proportion of classified grades for all the 5 min segments across the 1 h epoch.

The channel independent nature of our proposed system allows for the use of a reduced number of channels, as would be required in the widely used aEEG monitors. From table 7, it is clear that the performance using only two channels (F3-C3 and F4-C4) over the validation dataset is very similar to that obtained using all eight bipolar channels. This is not unexpected as HIE is a global injury. There may be advantages to a reduced montage, in particular for use with the common 2-channel aEEG machines. Nonetheless, other factors should also be considered. Although our proposed method is channel independent, future methods may incorporate spatial information such as connectivity measures. Visual assessment of the EEG may be more reliable when using a full montage, rather than just two channels. EEG is mostly used for seizure surveillance of neonates in the NICU. Multiple studies have demonstrated that full-montage EEG is superior to a reduced channel montage when detecting seizures, for example see [32, 33].

The proposed system uses a bandpass (derivative) filter and the log of the TFD, which is somewhat similar to the construction of the aEEG in that lower and higher frequencies are suppressed and amplitude is compressed by the log function. Similar in conception to the aEEG, the TFD transformation provides a level of abstraction—the aEEG by time-compression and the TFD by suppressing phase-differences between signal components [22, 28]. As much as there are similarities however, there are also many differences: the nonlinear filter of the aEEG differs to the forward-finite difference which approximates a derivative function; the aEEG has a log-linear scale, whereas the TFD uses the log of the absolute value; and probably most significant, the aEEG is a compressed time-domain signal whereas the TFD represents components in the time–frequency plane.

Within the validation dataset, we find a larger percentage of high-amplitude artefacts in the normal–mild epochs comparative to the %artefact in the other 3 grades ($P\lt0.001$), which may be associated with increased movement. This is likely due to the fact that these infants are firstly less encephalopathic and therefore more alert, and secondly less likely to be cooled with associated sedative medication. The potential danger with this finding is that an algorithm could use the very distinct characteristics of high-amplitude to help determine the grade. Movement artefacts are not generated by cortical activity and therefore should not aid classification of EEG grades. This is a similar problem to the issue of correctly classifying images based on text, such as copyright or watermarks, rather than the image itself. The proposed algorithm does not seem to suffer this same fate, as we found no difference (*P* = 0.784) in the %artefact between the correct- and incorrect-classification epochs. This could be because the algorithm was trained on clean data, with minimal artefacts. It will be important for the next iteration of algorithms to develop a system to remove artefacts from EEG, and for the algorithm to cope with missing data segments, before training on a less curated data set.

The CNNs were developed specifically for two-dimensional (2D) image data. EEG is a time-domain signal that has important information present in both time and frequency domains. Representing EEG in the joint time–frequency domain is an appropriate transformation for the highly non-stationary signal, revealing the time-varying structure in a low signal-to-noise environment. Quadratic TFDs have being used in a variety of neonatal EEG applications [22, 28, 29], including EEG HIE-grading [7]. The 2D nature of the TFDs are a perfect fit for the 2D network structure of CNNs. We find better performance using the 2D CNN with the TFD compared to the performance of the 1D CNN using raw EEG data [13]. The flexibility of the 2D network allows for the extraction of features along the time-, frequency-, and joint time–frequency directions of the TFD, thus representing a more diverse feature set compared to time-domain alone.

Our first CNN approach [13] extracted feature maps from the raw EEG signal in the time domain. This approach improved on both the TFDfeat [7] and IBIfeat [11] methods, but was not as accurate as the GSVfeat [10] system. However, we found that transforming the EEG to the time–frequency domain and using 3 independent CNN structures to evaluate important convolutional features in the time, frequency, and time–frequency domain improved performance. This increase in accuracy may be due to the following: (1) the time–frequency representation will increase the signal-to-noise ratio as noise is typically spread across time and frequency whereas the signal is often localised [22], (2) although CNNs can be adjusted for 1-dimensional signals, they were original designed for 2-dimensional signals and therefore may be more suited to this domain, (3) the time–frequency distribution adds a level of abstraction to the signal representation, thus increasing generalization [28, 29], and (4) our proposed CNN can extract features associated with changes in the time, frequency, and time–frequency direction which adds an extra dimension to the network compared with just time-convolution only.

A CNN approach for neonatal EEG analysis has been shown to outperform feature-based methods in seizure detection [34, 35] and sleep staging [14]. Although requiring significant computational resources during training, once trained, the networks are computationally efficient [36] and can be easily implemented in hardware platforms with limited resources [36]. In comparison to feature-based methods, CNNs have been shown to be robust to noisy labels and increase in performance with increasing data, unlike feature-based methods [37]. CNNs can learn high-level features of the EEG, which in turn can provide greater robustness to intra-class variability compared to feature-based methods [34, 35].

## 6. Conclusion

We present a novel deep-learning approach for grading EEG HIE-abnormalities based on quadratic TFDs of newborn EEG. The CNN architecture can learn relevant time, frequency, and time–frequency features to classify the 4 EEG HIE-grades. The developed system achieved greater accuracy compared with the state-of-the-art EEG HIE-grading systems, without the need to create complex feature sets. The method is channel independent and works well with a 2-channel montage. Such an automated system could help assist around the clock clinical decision making for neonates with hypoxic-ischaemic encephalopathy.

## Data Availability

No new data were created or analysed in this study.
